# Characteristics of the plasmid-mediated colistin-resistance gene *mcr-1* in *Escherichia coli* isolated from a veterinary hospital in Shanghai

**DOI:** 10.3389/fmicb.2022.1002827

**Published:** 2022-10-28

**Authors:** Hongguang Lin, Wenxin Chen, Rushun Zhou, Jie Yang, Yong Wu, Jiaomei Zheng, Shuyue Fei, Guiting Wu, Zhiliang Sun, Jiyun Li, Xiaojun Chen

**Affiliations:** ^1^College of Veterinary Medicine, Hunan Agricultural University, Changsha, Hunan, China; ^2^Hunan Engineering Technology Research Center of Veterinary Drugs, Hunan Agricultural University, Changsha, Hunan, China; ^3^Hunan Provincial Institution of Veterinary Drug and Feed Control, Changsha, Hunan, China; ^4^Changsha Animal and Plant Disease Control Center, Changsha, Hunan, China

**Keywords:** pet hospital, colistin, *mcr-1*, *Escherichia coli*, multidrug-resistant bacteria

## Abstract

The mobile colistin-resistance *(mcr)-1* gene is primarily detected in *Enterobacteriaceae* species, such as *Escherichia coli* and *Salmonella enterica*, and represents a significant public health threat. Herein, we investigated the prevalence and characteristics of *mcr-1*-positive *E*. *coli* (MCRPEC) in hospitalized companion animals in a pet hospital in Shanghai, China, from May 2021 to July 2021. Seventy-nine non-duplicate samples were collected from the feces (*n* = 52) and wounds (*n* = 20) of cats and dogs and the surrounding hospital environment (*n* = 7). Seven MCRPEC strains, identified using screening assays and polymerase chain reaction, exhibited multidrug-resistant phenotypes in broth-microdilution and agar-dilution assays. Based in whole-genome sequencing and bioinformatics analyses, all seven isolates were determined to belong to sequence type (ST) 117. Moreover, the Incl2 plasmid was prevalent in these MCRPEC isolates, and the genetic environment of the seven *E*. *coli* strains was highly similar to that of *E*. *coli* SZ02 isolated from human blood. The isolates also harbored the β-lactamase gene *bla*_CTX-M-65_, and florfenicol resistance gene *floR*, among other resistance genes. Given that horizontal transfer occurred in all seven strains, *E*. *coli* plasmid transferability may accelerate the emergence of multidrug-resistant bacteria and may be transmitted from companion animals to humans. Therefore, the surveillance of MCRPEC isolates among companion animals should be strengthened.

## Introduction

Antibiotics have remained a key means of treating bacterial infections in humans and animals; however, bacterial resistance reduces the effectiveness of treatment, posing a serious threat to public health (Sun et al., 2020). In particular, the continued spread of multidrug-resistant (MDR) bacteria has caused high morbidity and mortality rates worldwide. In fact, an estimated 10 million people will die as a result of drug-resistant infections by 2050 (O’Neill, 2016). Antimicrobial resistance (AMR) and antibiotic-resistance genes (ARG) pose multi-faceted challenges and are among the greatest threats to human health in the 21st century (Torres et al., 2021). Meanwhile, colistin was once reintroduced as the last resort against MDR and carbapenem-resistant infections (Shen et al., 2019) and is, thus, included in the World Health Organization’s list of antibiotics of critical importance in human medicine (World Health Organization, 2019). Bacterial resistance to colistin is thought to be acquired by chromosomal point mutations (Carroll et al., 2019). Moreover, colistin-resistance mechanisms are related to modulation of the bacterial cell surface, including variations in LPS structure, or due to shedding of the capsular polysaccharides that bind, or trap, colistin (Olaitan et al., 2014). However, the first plasmid-mediated colistin-resistance gene, mobile colistin-resistance 1 (*mcr-1*), was first reported in China in 2015 (Liu et al., 2016), thereby breaking the “last line of defense,” and increasing the threat of ARGs to public health.

Several *mcr-1* variants (*mcr-1* to *mcr-1*0) have been identified (Hussein et al., 2021) with plasmid-mediated colistin-resistance genes having been reported in 47 countries as widely distributed in humans, animals, water, food, and the environment (Shen et al., 2020). *Escherichia coli* is the most prevalent species harboring *mcr*-positive isolates, followed by *Salmonella enterica* and *Klebsiella pneumoniae*. The *mcr-1* gene is widely distributed in three major plasmid types, namely, IncI2, IncHI2, and IncX4 (Nang et al., 2019). A previous study revealed that the tandem configuration of ISApl1-*mcr-1* with different components enables a more diverse genetic context for *mcr-1* (Yu et al., 2017). Meanwhile, comparative analyses of pMCR_1410 and pHNSHP45 revealed that conjugative plasmid transfer genes may be involved in interspecies plasmid transfer (Zhao and Zong, 2016), suggesting the potential spread of *mcr-1* to a more diverse bacterial pool.

Drug-resistant bacteria can spread from animals to humans through direct contact, the food chain, and the environment (World Health Organization, 2014). In recent years, *mcr-1* and its variants have primarily been detected in livestock such as pigs and cattle (Wang et al., 2019, 2021; Cheng et al., 2021; Liu et al., 2022), and increasingly in companion animals, in China (Wang et al., 2018; Zhang et al., 2019, 2021; Lei et al., 2021). In fact, *Mcr-1* has been found in 8.7% of *Enterobacteriaceae* isolates from companion animals in Beijing, and *mcr-1*-positive *E*. *coli* (MCRPEC) transmission between companion animals *via* close contact has been observed in a pet shop in Guangzhou (Zhang et al., 2016; Lei et al., 2017). Importantly, colistin is routinely used for the treatment of bacterial infections in both humans and companion animals, while MCRPEC strain transmission occurs readily *via* close contact. Thus, to assess the potential risk of transmitting resistant bacteria to humans, it is necessary to accurately determine whether companion animals host bacteria carrying the *mcr-1* gene. Therefore, in the current study, we investigate the prevalence and characteristics of MCRPEC isolates from companion animals at a typical pet hospital in Shanghai, China.

## Materials and methods

### Sample collection and bacterial identification

As a first-tier city in China, Shanghai has a large population of pet owners; therefore, it was selected as a representative city for this study, and a typical pet hospital in the city was selected. With the consent of the pet owners, 79 non-duplicate samples (environment: *n* = 7, fecal: *n* = 52, wounds: *n* = 20) were collected from 22 cats and 9 dogs and the general clinic environment between May 2021 and July 2021. To identify colistin-resistant gram-negative strains, the samples were enriched in Mueller–Hinton (MH) broth (Landbridge, Beijing, China) at 37°C for 24 h, without shaking; thereafter, they were inoculated on MacConkey agar (Landbridge, Beijing, China) supplemented with 2 mg/L colistin (Meilun Biotechnology Co. Ltd., Dalian) and 30 mg/L vancomycin (Meilun Biotechnology Co. ltd. Dalian) and cultured at 37°C for an additional 24 h. Single, pure colonies were subsequently selected and inoculated into 500 μl of fresh Luria-Bertani broth containing 2 mg/L colistin (Hu et al., 2022). The presence of *mcr* genes was confirmed using polymerase chain reaction (PCR) with primers that amplify *mcr-1* to *mcr-10* (Mentasti et al., 2021). PCR with 16S rDNA primers was used to identify the species, as described previously (Srivastava et al., 2008). The *mcr*-positive strains were stored at −80°C until further use.

### Antimicrobial susceptibility testing for MCRPEC

Broth-microdilution and agar-dilution assays were performed to determine the minimum inhibitory concentrations (MICs) of colistin and 10 antibiotics (cefotaxime, ceftiofur, gentamicin, amikacin, ciprofloxacin, meropenem, florfenicol, tetracycline, tigecycline, and piperacillin-tazobactam Meilun Biotechnology Co. Ltd., Dalian) as recommended by the Clinical and Laboratory Standards Institute (CLSI)^1^ and the European Committee on antimicrobial susceptibility testing (EUCAST).^2^ *Escherichia coli* ATCC25922 was used as the quality-control strain. According to a previous report, bacteria resistant to three or more antibiotics were considered to be MDR strains (Zhao et al., 2021).

### Plasmid conjugation assays

The transferability of the colistin-resistance gene *mcr-1* was determined using filter mating with sodium azide-resistant *E*. *coli* J53 as the recipient strain and the MCRPEC isolates as the donor. During the logarithmic growth phase, the recipient and donor strains were mixed in a volume ratio of 1:3. A sterilized 0.45 μm microporous filter membrane was applied to MH medium, and the mixture was spread onto the membrane. After culturing at 37°C for 12 h, transconjugants were selected on MacConkey agar containing 4 mg/L colistin and 200 mg/L sodium azide and confirmed using enterobacterial repetitive intergenic consensus (ERIC)-PCR and PCR analysis of *mcr-1* (Liu et al., 2020; Shafiq et al., 2022).

### Whole-genome sequencing and bioinformatics analysis

DNA was extracted from all *mcr-1*-positive isolates using the TIANamp Bacteria DNA kit (Tiangen Biotech Co. Ltd., Beijing, China), according to the manufacturer’s instructions. Whole-genome sequencing was performed by Annoroad Gene Technology (Beijing, China) on the NovaSeq 6,000 S4 sequencing platform with NovaSeq 6,000 S4 Reagent kit V1.5. Bacterial genome assembly was performed using the SPAdes software (version 3.11) (Bankevich et al., 2012), and the draft sequence was annotated using PATRIC (version 3.6.9)^3^ (Antonopoulos et al., 2019). The virulence factors and AMR genes of the strains were analyzed using the ResFinder and VirulenceFinder tools of the Center for Genomic Epidemiology.^4^ The relationship with MCRPEC was evaluated using core-genome alignments and phylogenetic trees, constructed using Parsnp with the neighbor-joining method, and visualized using the online tool iTOL 6.5.7^5^ (Hu et al., 2022). The genetic environment of *mcr-1* was investigated using the Easyfig 2.2.215 tool (Sullivan et al., 2011). The comparative genomic map was generated using the BRIG 0.9516 tool (Alikhan et al., 2011).

## Results

### MCRPEC isolation and genus identification

Colistin-resistant bacteria were detected in 56 of the 79 samples. Seven MCRPEC isolates were identified using PCR with multiple primers (dogs: *n* = 2, cats: *n* = 5; Table 1).

**Table 1:** Antimicrobial susceptibility profiles of 7 *mcr-1*-positive *Escherichia coli* (MCRPEC) isolates from companion animals at a pet hospital in Shanghai, China.

Isolate	Species	Source	Animal procedure	Minimum inhibitory concentration (mg/L)										
				MEM	CL	AMK	CIP	GEN	CTX	TGC	PTZ	FFC	CEF	TCY
L23cr-1	*E*. *coli*	Dog feces	Blood test	0.007	8	1	1	4	8	≤ 0.015	1	256	8	0.5
L25cr-2	*E*. *coli*	Cat feces	Sterilization operation	0.007	8	1	1	8	16	0.25	1	256	8	≤ 0.25
L27cr-1	*E*. *coli*	Cat feces	Soft tissue contusion	0.007	8	1	1	4	8	0.25	1	256	8	≤ 0.25
L30cr-1	*E*. *coli*	Cat feces	Sterilization operation	≤ 0.004	16	1	1	8	8	0.5	1	256	8	0.5
L36cr-1	*E*. *coli*	Cat feces	Medical checkup	0.007	16	1	1	8	8	0.25	1	256	8	≤ 0.25
L40cr	*E*. *coli*	Dog wound	Abdominal surgery	0.007	8	1	1	4	8	0.25	2	256	8	≤ 0.25
L52cr-1	*E*. *coli*	Cat feces	Sterilization operation	0.007	8	1	1	4	8	0.25	1	256	8	≤ 0.25

Minimum inhibitory concentrations indicating resistant isolates are shown in bold text. The drug resistance breaking points of MEM, TCY, and FFC were according to Clinical and Laboratory Standards Institute standard. The drug resistance breaking points of CL, AMK, CIP, GEN, CTX, TGC, CEF, and PTZ were according to the European Committee on antimicrobial susceptibility testing standard. AMK, amikacin; CEF, ceftiofur; CIP, ciprofloxacin; CL, colistin; CTX, cefotaxime; FFC, florfenicol; GEN, gentamicin; MEM, meropenem; PTZ, piperacillin-tazobactam; TCY, tetracycline; TGC, tigecycline.

### Antimicrobial susceptibility profiles

The resistance profiles of the seven MCRPEC isolates to various antimicrobials are shown in Table 1. All seven isolates exhibited multidrug resistance, including resistance to colistin, cefotaxime, and florfenicol. The MIC of florfenicol was 256 mg/L. Three isolates (L25cr-2, L30cr-1, and L36cr-1) were also resistant to gentamicin. All seven isolates were susceptible to amikacin, ciprofloxacin, tigecycline, piperacillin-tazobactam, and ceftiofur.

### Plasmid conjugation assay

Conjugation assay results revealed that the *mcr-1-*carrying plasmid from all seven isolates was successfully transferred to recipient *E*. *coli* J53 cells. After three repeated conjugation experiments, the transfer success rate reached 100% (Supplementary Table S1).

### Genomic epidemiology

**Figure 1:**
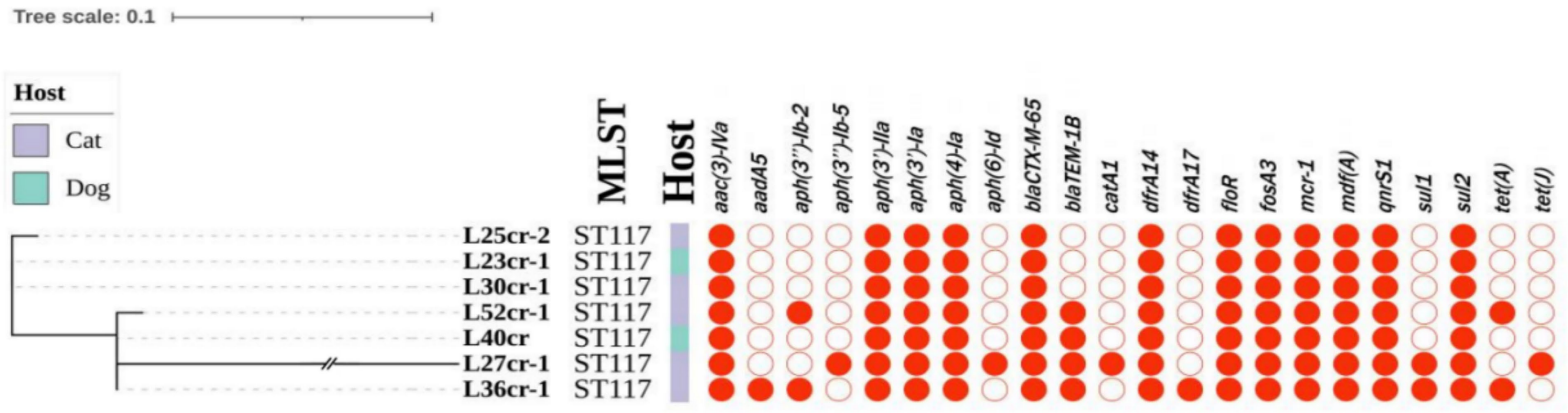
Phylogenetic tree (left) showing the genetic relationship, multi-locus sequence typing (MLST), and antimicrobial resistance genes of 7 *mcr-1*-positive *Escherichia coli* isolates from seven non-duplicate samples (feces and wounds) obtained from 5 cats and 2 dogs at a pet hospital in Shanghai.

**Figure 2:**
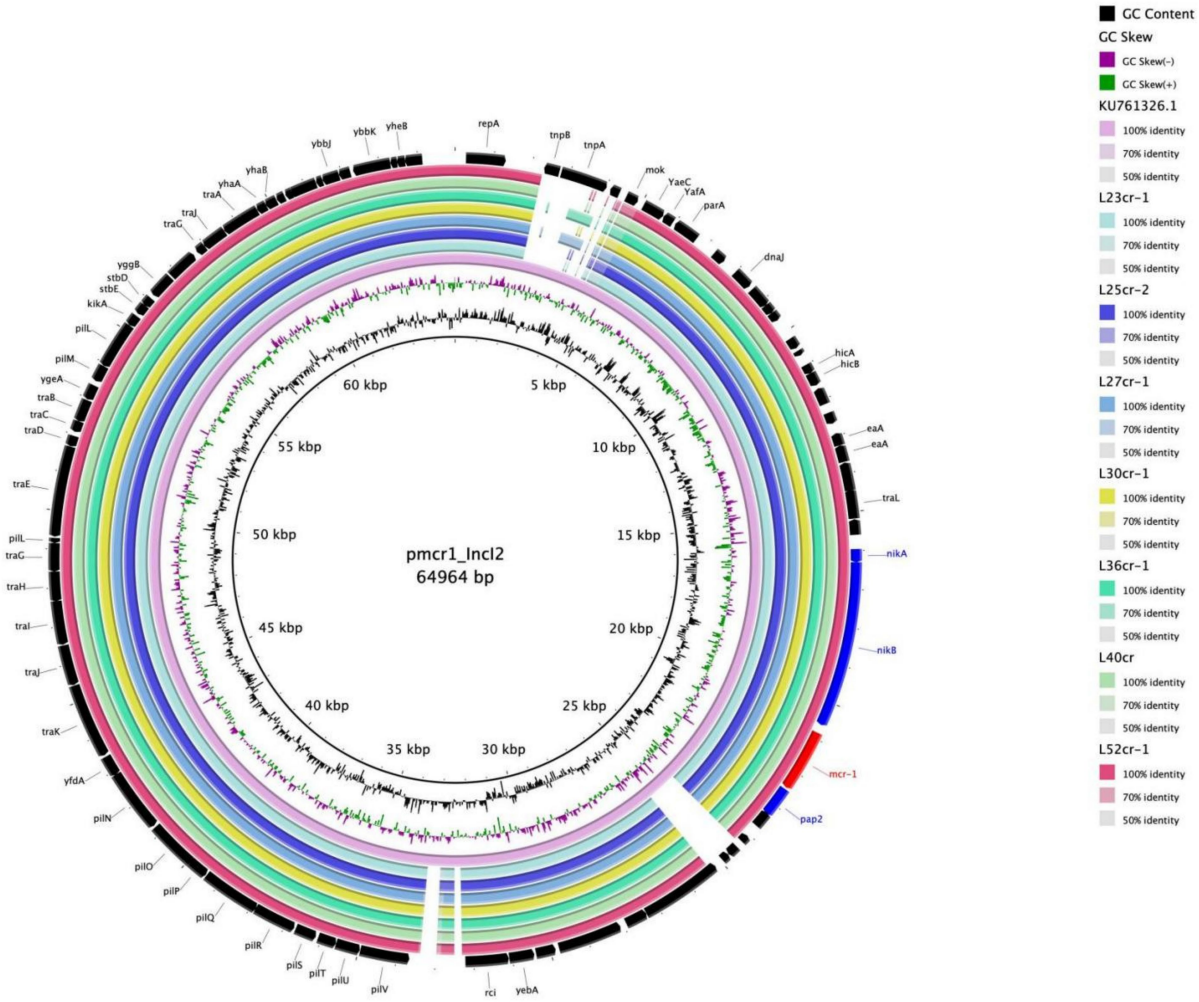
Multiple circular sequence alignments of reference plasmids with homologous overlapping strain groups identified in this study. The ring represents the corresponding plasmids shown in the legend. The reference plasmid is pmcr1_IncI2 (GenBank accession number: KU761326.1).

**Figure 3:**
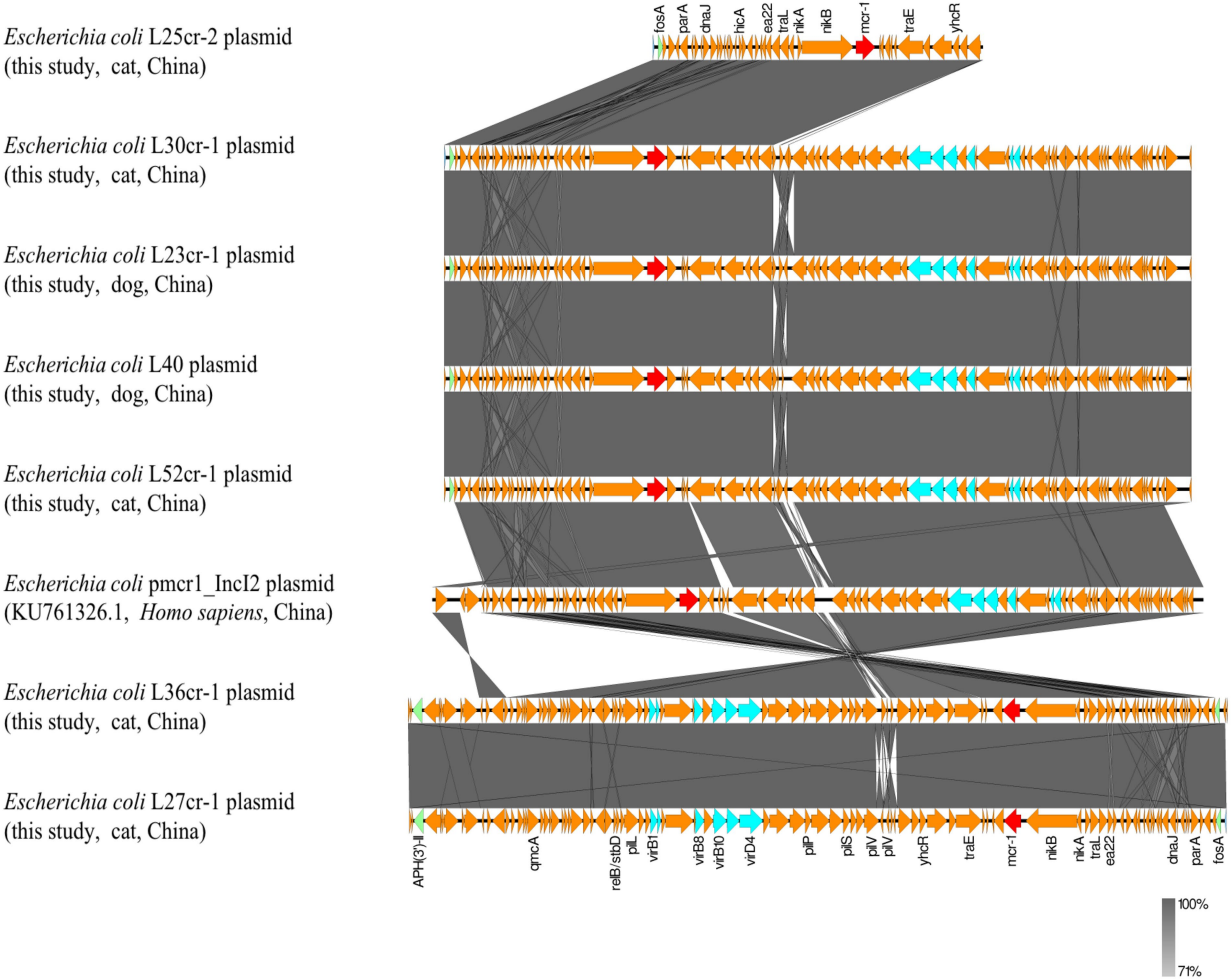
Genetic environment analysis of the 7 *mcr-1-*positive strains from samples obtained from a pet hospital in Shanghai, China, and reference strains. Arrows indicate the transcription direction. Shared regions with a high degree of sequence similarity are indicated in gray. Red arrows denote *mcr-1*, green arrows indicate other antibiotic-resistance genes, blue arrows indicate type IV secretion system genes, and orange arrows indicate other genes.

Multi-locus sequence typing assigned all seven isolates to the ST117 sequence type (ST; Figure 1). The genetic environment of the seven *E*. *coli* strains was highly similar to that of *E*. *coli* SZ02 isolated from human blood (GenBank accession number: KU761326.1; Figure 2). All strains were identified to have the same plasmid combination and the same serotype. The six *mcr-1-*positive plasmids were identified as Incl2 plasmids. However, the plasmids in L25cr-2 were difficult to characterize owing to their short sequences (Supplementary Table S1). All MCRPEC isolates harbored the common structure of nikA*-*nikB*-mcr-1-PAP2* (Figure 3) that is reportedly readily transferred horizontally to different plasmids (Shen et al., 2019, 2020). Mobile genomic elements (*ISKpn8*, *ISKpn19*, *ISKpn24*, *ISEc1*, *ISEc38*, *ISEc39*, *ISEc48*, *ISEc59*, *IS30*, *IS102*, *IS421*, *IS629*, *IS911*, *IS1006*, *ISSfl10*, *MITEEc1*, *cn_5813_IS911*, and *cn_3566_ISEc1*) co-existed in the seven MCRPEC isolates (Supplementary Table S1). However, no IS*Apl1* insertion sequences were observed upstream or downstream of *mcr-1*. Plasmid conjugative transfer genes (*traA*, *traD*, *traJ*, *traG*, *traE*, *traH*, *traL*, *pilL*, *pilU*, *pilR*, *pilQ*, *pilN*, *pilM*, *pilP*, *pilS*, *pilT*, and *pilV*) and those associated with the type IV secretion system (*virB1*, *virB2*, *virB8*, *virB10*, *virB11*, and *virD4*) were located in the *mcr*-bearing plasmids, facilitating *mcr-1* transfer to other bacteria.

### Distribution of AMR and virulence-associated genes among *mcr-1* isolates

Whole-genome sequencing analysis revealed that all MCRPEC strains carried additional resistance genes (Figure 1). In addition to *mcr-1*, aminoglycoside resistance genes [*aac(3)-IVa*, *aph(3*′*)-IIa*, *aph(3*′*)-Ia*, and *aph(4)-Ia*], a trimethoprim resistance gene (*dfrA14*), fosfomycin resistance gene (*fosA*), tigecycline resistance genes (*tet*(*A*) and *tet*(*J*)), and sulfonamide resistance genes (*sul1* and *sul2*) were present in the seven isolates. All strains contained the β-lactamase gene (*bla*_CTX-M-65_) and the florfenicol resistance gene (*floR*) (Figure 1).

The seven isolates were screened for virulence factor genes using the VirulenceFinder tool (version 2.0); the associated gene profiles are presented in Supplementary Figure S1. Many virulence genes, namely fimbrial-related genes (*papB/C/D/E/F/G/H/I/J/K* and *yfcV*), heme absorption–related genes (*chuT/U/V/W/Y*), a secretion system gene (*espL1*), and vacuolating autotransporter toxin gene (*vat*), as well as those encoding the siderophore receptor (*fyuA*), aerobactin synthetases (*iucA/B/C/D* and *iutA*), common pili (*ecpA/B/C/D/E/R*), and type I fimbriae (*fimA/B/C/D/E/F/G/H/I*) were present in all seven MCRPEC strains. However, tetracycline resistance genes [*tet*(*A*) and *tet*(*J*)], and *vat* were only isolated in the five cat samples.

## Discussion

Reports on the emergence of AMR, particularly colistin-resistance, are increasing worldwide (Shafiq et al., 2019). Meanwhile, the close contact between humans and companion animals may lead to bacterial horizontal transmission. However, the presence of ARGs in companion animals was not previously investigated in-depth (Chen et al., 2019). Nevertheless, *mcr-1* has previously been detected in companion animals in China, with an average detection rate of 6.9% (Zhang et al., 2016, 2021; Lei et al., 2017, 2021; Wang et al., 2018). *E*. *coli* is a common pathogen isolated from companion animals (Mathers et al., 2015). In this study, MCRPEC isolates were found in 7/79 samples collected from companion animals and a veterinary clinic environment. Meanwhile, colistin had not been used in the veterinary hospital. Moreover, the seven host animals, from which the MCRPEC isolates were obtained, were housed in the same hospital ward, suggesting that *mcr-1* (or the resistant *E*. *coli* strain harboring it) was circulating among these animals and their environment. This hypothesis was further supported by assessing the *E*. *coli* clonality from different dogs and cats.

Owing to the daily movement and living environment of companion animals, we suspect that the presence of *mcr-1* in this hospital originated from direct contact with humans, other animals, flies, or *via* ingestion of contaminated food sources. In fact, transfer of *mcr-1* from animals to humans was demonstrated by a MCRPEC case that was reportedly isolated from a human who had been in contact with farm animals, with no travel history (Izdebski et al., 2016). Moreover, all MCRPEC isolates were successfully transferred to *E*. *coli* J53 *via* conjugation in this study. Indeed, successful conjugation transfers have been reported for clinical, as well as environmental, samples in China (Christie, 2016; Yang et al., 2017; Liu et al., 2020). Given that *mcr-1* was of the same plasmid type and our conjugation transfer experiments were successful, we believe that horizontal gene transfer of *mcr-1*-harboring plasmids is the most likely transmission route among animals in the pet hospital. This transferability of *E*. *coli* plasmids may lead to accelerated ARG flow and promotion of MDR bacteria emergence.

MCRPEC isolates are typically resistant to multiple antibiotic classes (Donà et al., 2017). This was demonstrated in the current study, as *mcr-1* was found to co-exist with other important resistance genes [*aac*(*3*)*-IVa*, *bla*_CTX-M-65_, *fosA3*, and *floR*], which may be related to the use of other antibiotics (e.g., florfenicol and penicillin). In fact, all seven MCRPEC isolates were MDR isolates. Importantly, all antibiotics tested, herein, excluding florfenicol, are also commonly administered to humans, which provides high selection pressure for MDR bacteria. However, in this study, although the L36cr-1 isolate harbored several resistance genes, the host animal (a cat) was only 1 month old and had not yet received any antibiotics. Hence, this animal may have become infected with this isolate *via* nosocomial or community contact, as the host animal lived in a city and moved around frequently.

All seven MCRPEC isolates had lost the IS*Apl1* sequence on both sides of *mcr-1*, resulting in a structure lacking IS*Apl1*, which many further reduce chromosomal integration, thereby increasing the stability of *mcr-1* in plasmid vectors and facilitating its widespread dissemination (Du et al., 2020; Mohsin et al., 2021). This finding, combined with the plasmid conjugation assay results, suggests that other mobile genetic elements (such as the type IV secretion system, *pil*, and *ter*) and *mcr-1* may have played an important role in its horizontal transmission in the seven strains. In fact, all seven strains harbored a type IV secretion system gene. Plasmid-mediated conjugated antibiotic-resistance and virulence gene transfer between different bacterial genera requires the type IV secretion system, wherein type IV pili enhance conjugative plasmid transfer (Christie, 2016; Kohler et al., 2018). Hence, the type IV secretion system can lead to an explosive emergence of multidrug resistance among populations of clinically significant pathogens (Cascales and Christie, 2003). Therefore, the surveillance of bacteria containing type IV secretion systems must be emboldened.

In a previous study (Li et al., 2016), we found a similar segment (nikA-nikB-*mcr-1-pap2*) among plasmids of strains from both humans and animals, further supporting the hypothesis that *mcr-1* can readily spread between humans and companion animals. Furthermore, all plasmids had the same ST, indicating that *mcr-1* was likely derived from a single source, and suggesting the occurrence of *E*. *coli* clonal and vertical transmission in this area. The coexistence of horizontal and vertical transmission in these seven MCRPEC strains may increase the risk of *mcr-1* transmission, thereby posing a threat to human health.

This study had certain limitations, our experiment was limited to detecting the prevalence of *mcr-1* in a small area. Nevertheless, these results highlight the need for urgent implementation of a wide-range surveillance plan in other regions of China. Furthermore, other important emerging resistance genes in companion animals should be monitored to better quantify their risk to human health.

In summary, the observed spread of mcr-1 in companion animals could be attributable to the horizontal and vertical transmission of plasmids. We demonstrated that MCRPEC from companion animals represents a potential risk to human health by highlighting the possible transmission of *mcr-1*. This gene confers colistin-resistance to *E*. *coli*, thereby limiting clinical treatment options for humans. To prevent the spread of *mcr-1* from having a negative impact, minimizing the opportunities for *mcr-1*-harboring strains to infect and proliferate in humans is necessary. Therefore, surveillance programs should be implemented globally to monitor the prevalence of MCRPEC in companion animals, standards for rational antibiotic use in companion animals must be established, and pet owners must be encouraged to adhere strictly to veterinary-directed antibiotic regimens.

## Data availability statement

The genome assemblies of the seven MCRPEC strains were deposited in GenBank under the BioProject accession number PRJNA812731.

## Author contributions

XC and JL: conceptualization and methodology. HL, WC, and JZ: investigation. HL, RZ, and SF: data curation and formal analysis. HL: writing – original draft. XC, HL, ZS, JY, YW, and GW: writing – review and editing. All authors contributed to the article and approved the submitted version.

## Funding

This research was funded by the Hunan Provincial Natural Science Foundation of China, grant number 2021JJ40234, and the China Postdoctoral Science Foundation, grant number 2020M682569.

## Conflict of interest

The authors declare that the research was conducted in the absence of any commercial or financial relationships that could be construed as a potential conflict of interest.

## Publisher’s note

All claims expressed in this article are solely those of the authors and do not necessarily represent those of their affiliated organizations, or those of the publisher, the editors and the reviewers. Any product that may be evaluated in this article, or claim that may be made by its manufacturer, is not guaranteed or endorsed by the publisher.

## Supplementary material

The Supplementary material for this article can be found online at: https://www.frontiersin.org/articles/10.3389/fmicb.2022.1002827/full#supplementary-material

## Footnotes

^1^ https://clsi.org

^2^ https://eucast.org

^3^ https://patricbrc.org/

^4^ www.genomicepidemiology.org

^5^ https://itol.embl.de

## Abbreviation

AMK: amikacin
AMR: antimicrobial resistance
CEF: ceftiofur
CIP: ciprofloxacin
CL: colistin
CTX: cefotaxime
ERIC-PCR: enterobacterial repetitive intergenic consensus PCR
FFC: florfenicol
GEN: gentamicin
MCRPEC: *mcr-1-*positive *E*. *coli*
MDR: multidrug-resistant
MEM: meropenem
MH: Mueller–Hinton
MIC: minimum inhibitory concentration
PCR: polymerase chain reaction
PTZ: piperacillin-tazobactam
ST: sequence type
TCY: tetracycline
TGC: tigecycline.

## References

[ref1] Alikhan N. F. Petty N. K. Ben Zakour N. L. Beatson S. A. (2011). BLAST ring image generator (BRIG): simple prokaryote genome comparisons. BMC Genomics 12:402. doi: 10.1186/1471-2164-12-402, PMID: 21824423 PMC3163573

[ref2] Antonopoulos D. A. Assaf R. Aziz R. K. Brettin T. Bun C. Conrad N.. (2019). PATRIC as a unique resource for studying antimicrobial resistance. Brief. Bioinform. 20, 1094–1102. doi: 10.1093/bib/bbx083, PMID: 28968762 PMC6781570

[ref3] Bankevich A. Nurk S. Antipov D. Gurevich A. A. Dvorkin M. Kulikov A. S.. (2012). SPAdes: a new genome assembly algorithm and its applications to single-cell sequencing. J. Comput. Biol. 19, 455–477. doi: 10.1089/cmb.2012.0021, PMID: 22506599 PMC3342519

[ref5] Carroll L. M. Gaballa A. Guldimann C. Sullivan G. Henderson L. O. Wiedmann M. (2019). Identification of novel mobilized colistin resistance gene *mcr-9* in a multidrug-resistant, colistin-susceptible *Salmonella enterica* serotype typhimurium isolate. MBio 10, e00853–e00819. doi: 10.1128/mBio.00853-19, PMID: 31064835 PMC6509194

[ref6] Cascales E. Christie P. J. (2003). The versatile bacterial type IV secretion systems. Nat. Rev. Microbiol. 1, 137–149. doi: 10.1038/nrmicro753, PMID: 15035043 PMC3873781

[ref7] Chen Y. Liu Z. Zhang Y. Zhang Z. Lei L. Xia Z. (2019). Increasing prevalence of ESBL-producing multidrug resistance *Escherichia coli* from diseased pets in Beijing, China from 2012 to 2017. Front. Microbiol. 10:2852. doi: 10.3389/fmicb.2019.02852, PMID: 31921034 PMC6915038

[ref8] Cheng P. Yang Y. Cao S. Liu H. Li X. Sun J.. (2021). Prevalence and characteristic of swine-origin *mcr-1*-positive *Escherichia coli* in northeastern China. Front. Microbiol. 12:712707. doi: 10.3389/fmicb.2021.712707, PMID: 34354696 PMC8329492

[ref9] Christie P. J. (2016). The mosaic type IV secretion systems. EcoSal Plus 7. doi: 10.1128/ecosalplus.ESP-0020-2015, PMID: 27735785 PMC5119655

[ref10] Donà V. Bernasconi O. J. Pires J. Collaud A. Overesch G. Ramette A.. (2017). Heterogeneous genetic location of *mcr-1* in colistin-resistant *Escherichia coli* isolates from humans and retail chicken meat in Switzerland: emergence of *mcr-1*-carrying IncK2 plasmids. Antimicrob. Agents Chemother. 61, e01245–e01217. doi: 10.1128/AAC.01245-17, PMID: 28848010 PMC5655086

[ref11] Du C. Feng Y. Wang G. Zhang Z. Hu H. Yu Y.. (2020). Co-occurrence of the *mcr-1*.*1* and *mcr-3*.*7* genes in a multidrug-resistant *Escherichia coli* isolate from China. Infect. Drug Resist. 13, 3649–3655. doi: 10.2147/IDR.S268787, PMID: 33116684 PMC7585518

[ref12] Hu J. Yang J. Chen W. Liu Z. Zhao Q. Yang H.. (2022). Prevalence and characteristics of *mcr-1*-producing *Escherichia coli* in three kinds of poultry in Changsha China. Front. Microbiol. 13:840520. doi: 10.3389/fmicb.2022.840520, PMID: 35464934 PMC9021793

[ref13] Hussein N. H. Al-Kadmy I. Taha B. M. Hussein J. D. (2021). Mobilized colistin resistance (*mcr*) genes from 1 to 10: a comprehensive review. Mol. Bio. Rep. 48, 2897–2907. doi: 10.1007/s11033-021-06307-y, PMID: 33839987

[ref14] Izdebski R. Baraniak A. Bojarska K. Urbanowicz P. Fiett J. Pomorska-Wesołowska M.. (2016). Mobile *mcr-1*-associated resistance to colistin in Poland. J. Antimicrob. Chemother. 71, 2331–2333. doi: 10.1093/jac/dkw261, PMID: 27330064

[ref15] Kohler V. Goessweiner-Mohr N. Aufschnaiter A. Fercher C. Probst I. Pavkov-Keller T.. (2018). TraN: a novel repressor of an *enterococcus* conjugative type IV secretion system. Nucleic Acids Res. 46, 9201–9219. doi: 10.1093/nar/gky671, PMID: 30060171 PMC6158623

[ref16] Lei L. Wang Y. He J. Cai C. Liu Q. Yang D.. (2021). Prevalence and risk analysis of mobile colistin resistance and extended-spectrum β-lactamase genes carriage in pet dogs and their owners: a population based cross-sectional study. Emerg. Microbes Infect. 10, 242–251. doi: 10.1080/22221751.2021.1882884, PMID: 33502946 PMC7889244

[ref17] Lei L. Wang Y. Schwarz S. Walsh T. R. Ou Y. Wu Y.. (2017). *Mcr-1* in *Enterobacteriaceae* from companion animals, Beijing, China, 2012–2016. Emerg. Infect. Dis. 23, 710–711. doi: 10.3201/eid2304.161732, PMID: 28322714 PMC5367392

[ref18] Li A. Yang Y. Miao M. Chavda K. D. Mediavilla J. R. Xie X.. (2016). Complete sequences of *mcr-1*-harboring plasmids from extended-spectrum-β-lactamase- and carbapenemase-producing *Enterobacteriaceae*. Antimicrob. Agents Chemother. 60, 4351–4354. doi: 10.1128/AAC.00550-16, PMID: 27090180 PMC4914624

[ref19] Liu Y. Y. Wang Y. Walsh T. R. Yi L. X. Zhang R. Spencer J.. (2016). Emergence of plasmid-mediated colistin resistance mechanism *mcr-1* in animals and human beings in China: a microbiological and molecular biological study. Lancet Infect. Dis. 16, 161–168. doi: 10.1016/S1473-3099(15)00424-7, PMID: 26603172

[ref20] Liu Z. Wang K. Zhang Y. Xia L. Zhao L. Guo C.. (2022). High prevalence and diversity characteristics of *blaNDM*, *mcr*, and *Bla* ESBLs harboring multidrug-resistant *Escherichia coli* from chicken, pig, and cattle in China. Front. Cell. Infect. Microbiol. 11:755545. doi: 10.3389/fcimb.2021.755545, PMID: 35198455 PMC8859839

[ref21] Liu F. Zhang R. Yang Y. Li H. Wang J. Lan J.. (2020). Occurrence and molecular characteristics of *mcr-1*-positive *Escherichia coli* from healthy meat ducks in Shandong Province of China. Animals 10:1299. doi: 10.3390/ani10081299, PMID: 32751361 PMC7459970

[ref22] Mathers A. J. Peirano G. Pitout J. D. (2015). The role of epidemic resistance plasmids and international high-risk clones in the spread of multidrug-resistant *Enterobacteriaceae*. Clin. Microbiol. Rev. 28, 565–591. doi: 10.1128/CMR.00116-14, PMID: 25926236 PMC4405625

[ref23] Mentasti M. David S. Sands K. Khan S. Davies L. Turner L.. (2021). Rapid detection and differentiation of mobile colistin resistance (*mcr-1* to *mcr-10*) genes by real-time PCR and melt-curve analysis. J. Hosp. Infect. 110, 148–155. doi: 10.1016/j.jhin.2021.01.010, PMID: 33485969

[ref24] Mohsin M. Hassan B. Martins W. Li R. Abdullah S. Sands K.. (2021). Emergence of plasmid-mediated tigecycline resistance *tet(X4)* gene in *Escherichia coli* isolated from poultry, food and the environment in South Asia. Sci. Total Environ. 787:147613. doi: 10.1016/j.scitotenv.2021.147613, PMID: 33992939

[ref25] Nang S. C. Li J. Velkov T. (2019). The rise and spread of mcr plasmid-mediated polymyxin resistance. Crit. Rev. Microbiol. 45, 131–161. doi: 10.1080/1040841X.2018.1492902, PMID: 31122100 PMC6625916

[ref26] O’Neill J. (2016). Tackling Drug-Resistant Infections Globally: Final Report and Recommendations Government of the United Kingdom.

[ref27] Olaitan A. O. Morand S. Rolain J. M. (2014). Mechanisms of polymyxin resistance: acquired and intrinsic resistance in bacteria. Front. Microbiol. 5:643. doi: 10.3389/fmicb.2014.00643, PMID: 25505462 PMC4244539

[ref28] Shafiq M. Huang J. Ur Rahman S. Shah J. M. Chen L. Gao Y.. (2019). High incidence of multidrug-resistant *Escherichia coli* coharboring *mcr-1* and *blaCTX-M-15* recovered from pigs. Infect. Drug Resist. 12, 2135–2149. doi: 10.2147/IDR.S209473, PMID: 31410033 PMC6643958

[ref29] Shafiq M. Rahman S. U. Bilal H. Ullah A. Noman S. M. Zeng M.. (2022). Incidence and molecular characterization of ESBL-producing and colistin-resistant *Escherichia coli* isolates recovered from healthy food-producing animals in Pakistan. J. Appl. Microbiol. Adv. 133, 1169–1182. doi: 10.1111/jam.15469, PMID: 35094463

[ref30] Shen Y. Lv Z. Yang L. Liu D. Ou Y. Xu C.. (2019). Integrated aquaculture contributes to the transfer of *mcr-1* between animals and humans via the aquaculture supply chain. Environ. Int. 130:104708. doi: 10.1016/j.envint.2019.03.056, PMID: 31202027

[ref31] Shen Y. Zhang R. Schwarz S. Wu C. Shen J. Walsh T. R.. (2020). Farm animals and aquaculture: significant reservoirs of mobile colistin resistance genes. Environ. Microbiol. 22, 2469–2484. doi: 10.1111/1462-2920.14961, PMID: 32114703

[ref32] Srivastava S. Singh V. Kumar V. Verma P. C. Srivastava R. Basu V.. (2008). Identification of regulatory elements in 16S rRNA gene of *Acinetobacter* species isolated from water sample. Bioinformation 3, 173–176. doi: 10.6026/97320630003173, PMID: 19238242 PMC2639665

[ref33] Sullivan M. J. Petty N. K. Beatson S. A. (2011). Easyfig: a genome comparison visualizer. Bioinformatics 27, 1009–1010. doi: 10.1093/bioinformatics/btr039, PMID: 21278367 PMC3065679

[ref34] Sun C. Cui M. Zhang S. Liu D. Fu B. Li Z.. (2020). Genomic epidemiology of animal-derived tigecycline-resistant *Escherichia coli* across China reveals recent endemic plasmid-encoded *tet(X4)* gene. Commun. Biol. 3:412. doi: 10.1038/s42003-020-01148-0, PMID: 32737421 PMC7395754

[ref36] Torres R. T. Cunha M. V. Araujo D. Ferreira H. Fonseca C. Palmeira J. D. (2021). Emergence of colistin resistance genes (*mcr-1*) in *Escherichia coli* among widely distributed wild ungulates. Environ. Pollut. 291:118136. doi: 10.1016/j.envpol.2021.118136, PMID: 34530238

[ref39] Wang Z. Fu Y. Schwarz S. Yin W. Walsh T. R. Zhou Y.. (2019). Genetic environment of colistin resistance genes *mcr-1* and *mcr-3* in *Escherichia coli* from one pig farm in China. Vet. Microbiol. 230, 56–61. doi: 10.1016/j.vetmic.2019.01.011, PMID: 30827405

[ref40] Wang J. Huang X. Y. Xia Y. B. Guo Z. W. Ma Z. B. Yi M. Y.. (2018). Clonal spread of *Escherichia coli* ST93 carrying *mcr-1*-harboring IncN1-IncHI2/ST3 plasmid among companion animals. China. Front. Microbiol. 9:2989. doi: 10.3389/fmicb.2018.02989, PMID: 30564223 PMC6288184

[ref41] Wang X. Zhai Z. Zhao X. Zhang H. Jiang H. Wang X.. (2021). Occurrence and characteristics of *Escherichia coli mcr-1*-like in rabbits in Shandong. China. Vet. Med. Sci. 7, 219–225. doi: 10.1002/vms3.340, PMID: 33012114 PMC7840214

[ref42] World Health Organization Antimicrobial resistance: global report on surveillance. Geneva: World Health Organization (2014).

[ref43] World Health Organization Critically important antimicrobials for human medicine (WHO CIA list). Geneva: World Health Organization (2019).

[ref44] Yang D. Qiu Z. Shen Z. Zhao H. Jin M. Li H.. (2017). The occurrence of the colistin resistance gene *mcr-1* in the Haihe River (China). Int. J. Environ. Res. Public Health 14:576. doi: 10.3390/ijerph14060576, PMID: 28555063 PMC5486262

[ref45] Yu C. Y. Ang G. Y. Chong T. M. Chin P. S. Ngeow Y. F. Yin W. F.. (2017). Complete genome sequencing revealed novel genetic contexts of the *mcr-1* gene in *Escherichia coli* strains. J. Antimicrob. Chemother. 72, 1253–1255. doi: 10.1093/jac/dkw541, PMID: 28031273

[ref46] Zhang X. F. Doi Y. Huang X. Li H. Y. Zhong L. L. Zeng K. J.. (2016). Possible transmission of *mcr-1*-harboring *Escherichia coli* between companion animals and human. Emerg. Infect. Dis. 22, 1679–1681. doi: 10.3201/eid2209.160464, PMID: 27191649 PMC4994340

[ref47] Zhang Z. Lei L. Zhang H. Dai H. Song Y. Li L.. (2021). Molecular investigation of *Klebsiella pneumoniae* from clinical companion animals in Beijing, China, 2017–2019. Pathogens. 10:271. doi: 10.3390/pathogens10030271, PMID: 33673656 PMC7997213

[ref48] Zhang P. Wang J. Wang X. Bai X. Ma J. Dang R.. (2019). Characterization of five *Escherichia coli* isolates co-expressing ESBL and *mcr-1* resistance mechanisms from different origins in China. Front. Microbiol. 10:1994. doi: 10.3389/fmicb.2019.01994, PMID: 31555232 PMC6727855

[ref49] Zhao Q. Berglund B. Zou H. Zhou Z. Xia H. Zhao L.. (2021). Dissemination of *blaNDM-5* via IncX3 plasmids in carbapenem-resistant *Enterobacteriaceae* among humans and in the environment in an intensive vegetable cultivation area in eastern China. Environ. Pollut. 273:116370. doi: 10.1016/j.envpol.2020.116370, PMID: 33460870

[ref50] Zhao F. Zong Z. (2016). *Kluyvera ascorbata* strain from hospital sewage carrying the *mcr-1* colistin resistance gene. Antimicrobiol. Agents Chemother. 60, 7498–7501. doi: 10.1128/AAC.01165-16, PMID: 27671069 PMC5119035

