# Supplementary material for: Characteristics of the plasmid-mediated colistin-resistance gene mcr-1 in Escherichia coli isolated from a veterinary hospital in Shanghai
Source: Front Microbiol. 2022 Oct 28;13:1002827. doi: 10.3389/fmicb.2022.1002827 (PMC9650080; doi:10.3389/fmicb.2022.1002827)
Supplement: Supplementary file 1 [file Table_1.DOCX]

Supplementary Material

**Table S1.** Characteristics and antimicrobial resistance properties of the seven *mcr-1*-positive *Escherichia coli* isolates identified in this study.

| Strain | ST | Transferable (+/-) | *mcr-1* plasmid type | Antimicrobial resistance  genes | Virulence genes | Serotype | **Plasmid** | MGE |
| --- | --- | --- | --- | --- | --- | --- | --- | --- |
| L23cr-1 | ST117 | + | Incl2 | *aac(3)-IVa, aph(3')-IIa, aph(3')-Ia, aph(4)-Ia,blaCTX-M-65,dfrA14,*  *floR, fosA3, mcr-1, mdf(A), qnrS1, sul2* | *aslA, chuT, chuU, chuV, chuW, chuY, entB, entC, entD, entE, entF, entS, espL1, fdeC, fepA, fepB, fepC, fepD, fepG, fes, fimA, fimB, fimC, fimD, fimE, fimF, fimG, fimH, fimI, fyuA, gspC, gspD, gspE, gspF, gspG, gspH, gspI, gspJ, gspK, gspL, gspM, iroB, iroC, iroD, iroE, iroN, irp1, irp2, iucA, iucB, iucC, iucD, iutA, ompA, papB, papC, papD, papE, papF, papG, papH, papI, papJ, papK, pic, shuA, shuS, shuX, yagV/ecpE, yagW/ecpD, yagX/ecpC, yagY/ecpB, yagZ/ecpA, ybtA, ybtE, ybtP, ybtQ, ybtS, ybtT, ybtU, ybtX, ykgK/ecpR* | O161, H4 | IncI2, IncFIC(FII), IncFIB(AP001918), IncHI2A, IncHI2, Col156, ColRNAI, Col(MG828) | *ISKpn8, ISKpn19, ISKpn24, ISEc1, ISEc17, ISEc38, ISEc39, ISEc48, ISEc59, IS3, IS30, IS102, IS421, IS629, IS911, IS1006, IS5075, ISSfl10, MITEEc1, cn_5813_IS911, cn_3566_ISEc1* |
| L25cr-2 | ST117 | + | NA | *aac(3)-IVa, aph(3')-IIa, aph(3')-Ia, aph(4)-Ia,blaCTX-M-65,dfrA14,*  *floR, fosA3, mcr-1, mdf(A), qnrS1, sul2* | *aslA, chuT, chuU, chuV, chuW, chuY, entB, entC, entD, entE, entF, entS, espL1, fdeC, fepA, fepB, fepC, fepD, fepG, fes, fimA, fimB, fimC, fimD, fimE, fimF, fimG, fimH, fimI, fyuA, gspC, gspD, gspE, gspF, gspG, gspH, gspI, gspJ, gspK, gspL, gspM, iroB, iroC, iroD, iroE, iroN, irp1, irp2, iucA, iucB, iucC, iucD, iutA, ompA, papB, papC, papD, papE, papF, papG, papH, papI, papJ, papK, pic, shuA, shuS, shuX, yagV/ecpE, yagW/ecpD, yagX/ecpC, yagY/ecpB, yagZ/ecpA, ybtA, ybtE, ybtP, ybtQ, ybtS, ybtT, ybtU, ybtX, ykgK/ecpR* | O161, H4 | IncI2, IncFIC(FII), IncFIB(AP001918), IncHI2A, IncHI2, Col156, ColRNAI, Col(MG828) | *ISKpn8, ISKpn19, ISKpn24, ISEc1, ISEc17, ISEc38, ISEc39, ISEc48, ISEc59, IS3, IS30, IS102, IS421, IS629, IS911, IS1006, IS5075, ISSfl10, MITEEc1, cn_5813_IS911, cn_3209_ISEc1* |
| L27cr-1 | ST117 | + | Incl2 | *aac(3)-IVa,, aph(3'')-Ib, aph(3')-IIa, aph(3')-Ia, aph(4)-Ia, aph(6)-Id,*  *blaCTX-M-65, blaTEM-1B, catA1, dfrA14, floR, fosA3*  *mcr-1, mdf(A), qnrS1, sul1, sul2, tet(J)* | *aslA, chuT, chuU, chuV, chuW, chuY, entB, entC, entD, entE, entF, entS, espL1, fdeC, fepA, fepB, fepC, fepD, fepG, fes, fimA, fimB, fimC, fimD, fimE, fimF, fimG, fimH, fimI, fyuA, gspC, gspD, gspE, gspF, gspG, gspH, gspI, gspJ, gspK, gspL, gspM, iroB, iroC, iroD, iroE, iroN, irp1, irp2, iucA, iucB, iucC, iucD, iutA, ompA, papB, papC, papD, papE, papF, papG, papH, papI, papJ, papK, pic, shuA, shuS, shuX, vat, yagV/ecpE, yagW/ecpD, yagX/ecpC, yagY/ecpB, yagZ/ecpA, ybtA, ybtE, ybtP, ybtQ, ybtS, ybtT, ybtU, ybtX, ykgK/ecpR* | O161, H4 | IncI2, IncFIC(FII), IncFIB(AP001918), IncHI2A, IncHI2, Col156, ColRNAI, Col(MG828) | *ISKpn8, ISKpn19, ISKpn24, ISEc1, ISEc38, ISEc39, ISEc48, ISEc59, IS30, IS102, IS421, IS629, IS911, IS1006, ISSfl10, MITEEc1, cn_5813_IS911, cn_3566_ISEc1* |
| L30cr-1 | ST117 | + | Incl2 | *aac(3)-IVa, aph(3')-IIa,aph(3')-Ia, aph(4)-Ia,blaCTX-M-65,dfrA14,*  *floR, fosA3, mcr-1, mdf(A), qnrS1, sul2* | *aslA, chuT, chuU, chuV, chuW, chuY, entB, entC, entD, entE, entF, entS, espL1, fdeC, fepA, fepB, fepC, fepD, fepG, fes, fimA, fimB, fimC, fimD, fimE, fimF, fimG, fimH, fimI, fyuA, gspC, gspD, gspE, gspF, gspG, gspH, gspI, gspJ, gspK, gspL, gspM, iroB, iroC, iroD, iroE, iroN, irp1, irp2, iucA, iucB, iucC, iucD, iutA, ompA, papB, papC, papD, papE, papF, papG, papH, papI, papJ, papK, pic, shuA, shuS, shuX, yagV/ecpE, yagW/ecpD, yagX/ecpC, yagY/ecpB, yagZ/ecpA, ybtA, ybtE, ybtP, ybtQ, ybtS, ybtT, ybtU, ybtX, ykgK/ecpR* | O161, H4 | IncI2, IncFIC(FII), IncFIB(AP001918), IncHI2A, IncHI2, Col156, ColRNAI, Col(MG828) | *ISKpn8, ISKpn19, ISKpn24, ISEc1, ISEc38, ISEc39, ISEc48, ISEc59, IS3, IS30, IS102, IS421, IS629, IS911, IS1006, IS5075, ISSfl10, MITEEc1, cn_5813_IS911, cn_3566_ISEc1* |
| L36cr-1 | ST117 | + | Incl2 | *aac(3)-IVa, aadA5, aph(3'')-Ib, aph(3')-IIa, aph(3')-Ia, aph(4)-Ia*  *blaTEM-1B, blaCTX-M-65, dfrA14, floR, fosA3, mcr-1*  *mdf(A), qnrS1, sul1, sul2, tet(A)* | *aslA, chuT, chuU, chuV, chuW, chuY, entB, entC, entD, entE, entF, entS, espL1, fdeC, fepA, fepB, fepC, fepD, fepG, fes, fimA, fimB, fimC, fimD, fimE, fimF, fimG, fimH, fimI, fyuA, gspC, gspD, gspE, gspF, gspG, gspH, gspI, gspJ, gspK, gspL, gspM, iroB, iroC, iroD, iroE, iroN, irp1, irp2, iucA, iucB, iucC, iucD, iutA, ompA, papB, papC, papD, papE, papF, papG, papH, papI, papJ, papK, pic, shuA, shuS, shuX, vat, yagV/ecpE, yagW/ecpD, yagX/ecpC, yagY/ecpB, yagZ/ecpA, ybtA, ybtE, ybtP, ybtQ, ybtS, ybtT, ybtU, ybtX,ykgK/ecpR* | O161, H4 | IncI2, IncFIC(FII), IncFIB(AP001918), IncHI2A, IncHI2, Col156, ColRNAI, Col(MG828) | *ISKpn8, ISKpn19, ISKpn24, ISEc1, ISEc17, ISEc38, ISEc39, ISEc48, ISEc59, IS3, IS30, IS102, IS421, IS629, IS911, IS1006, IS5075, ISSfl10, MITEEc1, cn_5813_IS911, ISEc1, cn_3209_ISEc1* |
| L40cr | ST117 | + | Incl2 | *aac(3)-IVa, aph(3')-IIa, aph(3')-Ia, aph(4)-Ia, blaTEM-1B*  *blaCTX-M-65, dfrA14, dfrA17, floR, fosA3, mcr-1, mdf(A)*  *qnrS1, sul2* | *aslA, chuT, chuU, chuV, chuW, chuY, entB, entC, entD, entE, entF, entS, espL1, fdeC, fepA, fepB, fepC, fepD, fepG, fes, fimA, fimB, fimC, fimD, fimE, fimF, fimG, fimH, fimI, fyuA, gspC, gspD, gspE, gspF, gspG, gspH, gspI, gspJ, gspK, gspL, gspM, iroB, iroC, iroD, iroE, iroN, irp1, irp2, iucA, iucB, iucC, iucD, iutA, ompA, papB, papC, papD, papE, papF, papG, papH, papI, papJ, papK, pic, shuA, shuS, shuX, yagV/ecpE, yagW/ecpD, yagX/ecpC, yagY/ecpB, yagZ/ecpA, ybtA, ybtE, ybtP, ybtQ, ybtS, ybtT, ybtU, ybtX, ykgK/ecpR* | O161, H4 | IncI2, IncFIC(FII), IncFIB(AP001918), IncHI2A, IncHI2, Col156, ColRNAI, Col(MG828) | *ISKpn8, ISKpn19, ISKpn24, ISEc1, ISEc17, ISEc38, ISEc39, ISEc48, ISEc59, IS3, IS30, IS102, IS421, IS629, IS911, IS1006, IS5075, ISSfl10, MITEEc1, cn_5813_IS911, ISEc1, cn_3209_ISEc1* |
| L52cr-1 | ST117 | + | Incl2 | *aac(3)-IVa, aph(3'')-Ib, aph(3')-IIa, aph(3')-Ia, aph(4)-Ia*  *blaTEM-1B, blaCTX-M-65, dfrA14, floR, fosA3, mcr-1*  *mdf(A), qnrS1, sul2, tet(A)* | *aslA, chuT, chuU, chuV, chuW, chuY, entB, entC, entD, entE, entF, entS, espL1, fdeC, fepA, fepB, fepC, fepD, fepG, fes, fimA, fimB, fimC, fimD, fimE, fimF, fimG, fimH, fimI, fyuA, gspC, gspD, gspE, gspF, gspG, gspH, gspI, gspJ, gspK, gspL, gspM, iroB, iroC, iroD, iroE, iroN, irp1, irp2, iucA, iucB, iucC, iucD, iutA, ompA, papB, papC, papD, papE, papF, papG, papH, papI, papJ, papK, pic, shuA, shuS, shuX, yagV/ecpE, yagW/ecpD, yagX/ecpC, yagY/ecpB, yagZ/ecpA, ybtA, ybtE, ybtP, ybtQ, ybtS, ybtT, ybtU, ybtX, ykgK/ecpR* | O161, H4 | IncI2, IncFIC(FII), IncFIB(AP001918), IncHI2A, IncHI2, Col156, ColRNAI, Col(MG828) | *ISKpn8, ISKpn19, ISKpn24, ISEC1, ISEc17, ISEc38, ISEc39, ISEc48, ISEc59, IS3, IS30, IS102, IS421, IS629, IS911, IS1006, IS5075, ISSfl10, MITEEc1, cn_5813_IS911, cn_3566_ISEc1* |

NA, not available; ST, sequence typing


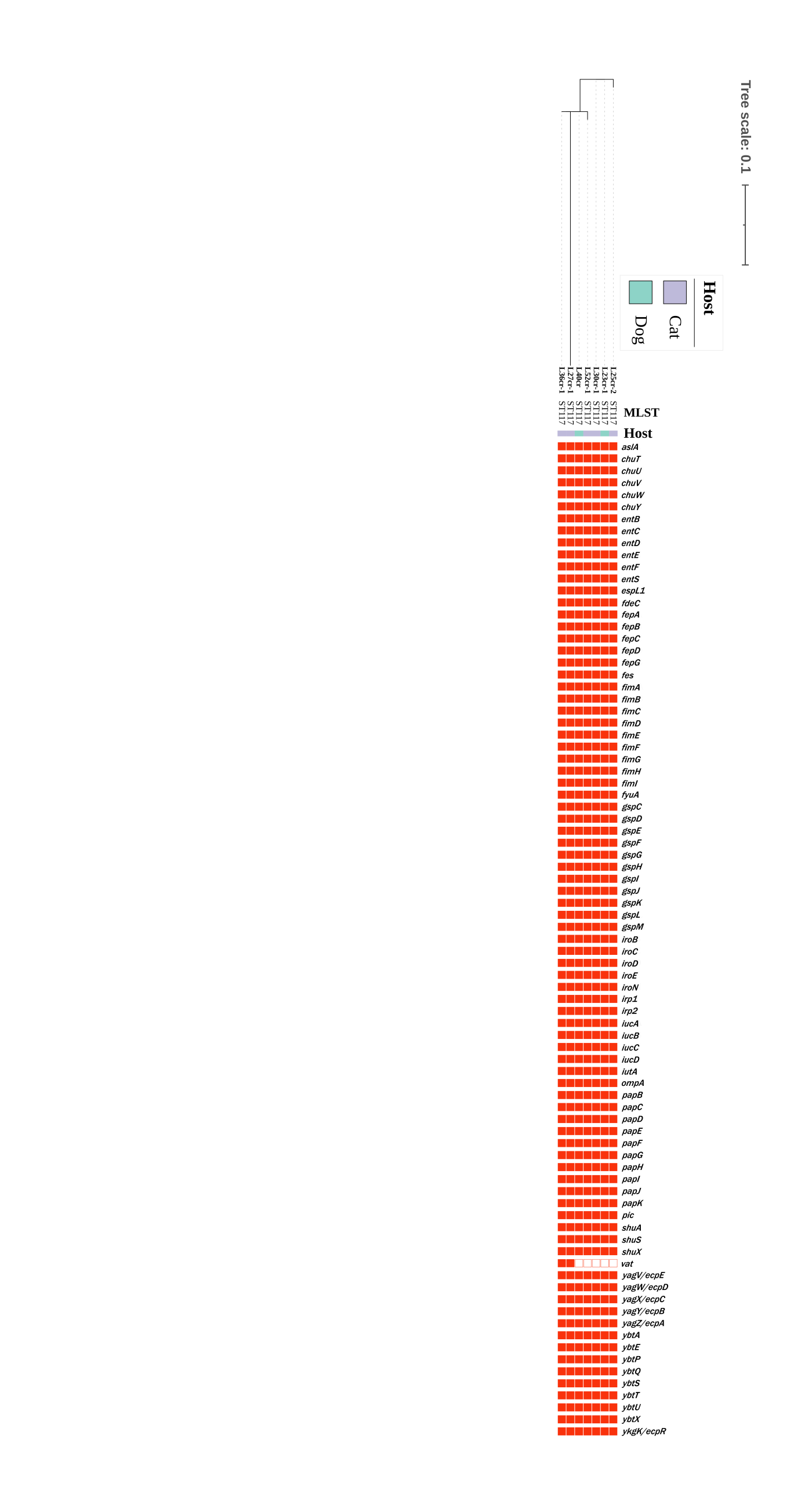


**Figure S1.** Phylogenetic tree showing the genetic relationship, multi-locus sequence typing (MLST) results, and virulence genes of *mcr-1*-positive *Escherichia coli* isolates from seven non-duplicate samples (feces and affected area) obtained from five cats and two dogs at a typical pet hospital in Shanghai.
